# Exploring cerebrospinal fluid metabolites, cognitive function, and brain atrophy: Insights from Mendelian randomization

**DOI:** 10.1515/med-2025-1237

**Published:** 2025-08-04

**Authors:** Qian Liu, Ling-bing Meng, Tian-qi Qi, Ya-Qing Ma, Guo-wei Liang

**Affiliations:** Department of Clinical Laboratory, Aerospace Center Hospital, Beijing, 100049, China; Cardiometabolic Medicine Center, National Clinical Research Center for Cardiovascular Diseases, Fuwai Hospital, National Center for Cardiovascular Diseases, Chinese Academy of Medical Sciences and Peking Union Medical College, Beijing, 100730, China; The Key Laboratory of Geriatrics, Beijing Institute of Geriatrics, Institute of Geriatric Medicine, Chinese Academy of Medical Sciences, Beijing Hospital/National Center of Gerontology of National Health Commission, Dong Dan, Beijing, 100730, China

**Keywords:** cerebrospinal fluid, metabolomics, cognition, brain atrophy, Mendelian randomization

## Abstract

Disruption of cerebrospinal fluid (CSF) metabolites affects brain function and cognition, potentially altering the brain structure. To elucidate the causal relationships between CSF metabolites and the neurological outcomes, we conducted a two-sample Mendelian randomization analysis. Genome-wide association data from 689 individuals of European descent provided exposure levels for metabolites, analyzed alongside gene associations for cognitive performance (*N*  =  257,841), brain atrophy measures (cortical surface area and thickness; *N*  =  51,665), and hippocampal volume (*N*  =  33,536). Our analysis identified 30 metabolites exhibiting causal associations with brain atrophy and cognitive function: 20 linked to cognition and 10 to structural atrophy. Notably, butyrate correlated strongest with the cortical surface area, bilirubin with the cortical thickness, methionine sulfoxide with the hippocampal volume, threonate with cognitive performance, while oxidized Cys-gly, *N*6-succinyladenosine, and *N*-acetylglucosamine were linked to fluid intelligence, prospective memory, and reaction time, respectively. Pathway analyses revealed that butanoate and niacinamide/niacin ester metabolism are significantly associated with brain atrophy and cognitive performance. These findings position CSF metabolites as promising therapeutic targets for neurodegenerative diseases, providing a causal framework to prioritize interventions. Experimental studies building on this genetic evidence hold potential to accelerate the development of mechanism-driven therapies targeting metabolic pathways in neurodegeneration.

## 1 Introduction

With increasing life expectancy, dementia has emerged as a significant global health challenge, with an estimated 150 million people projected to be affected by 2050 [1]. The brain starts changing years before clinical symptoms appear [2]. Early biomarkers help in studying the pathological mechanisms and potential prevention strategies.

In recent years, metabolomics research has revealed new insights into complex diseases such as diabetes, obesity, cancer, and Alzheimer’s disease (AD) [3,4,5,6], elucidating their disease mechanisms through the identification of relevant metabolites and discovering potential biomarkers for diagnosis and prognosis. Although most studies focus on blood or urine samples, cerebrospinal fluid (CSF) is particularly crucial for psychiatric and neurological disorders [7]. CSF can directly reflect physiological changes in the central nervous system. For example, in AD, CSF is an important source of biomarkers like amyloid-beta and phosphorylated tau [8]. A wealth of observational cohort studies have investigated the relationship between metabolites and brain structure and cognition, suggesting that these metabolites may have neuroprotective, anti-inflammatory, or other effects [9,10,11].

Due to the tendency for observational studies to encounter reverse causation and confounding effects, we employed Mendelian randomization (MR) using genetic variants as instrumental variables (IVs) based on Mendel’s law to explore causal relationships between exposure and outcome [12]. Cognitive function and brain atrophy markers are considered key features of AD and other forms of dementia [13,14], and a larger number of cases may be more suitable for dissecting potential causal relationships between CSF metabolites and dementia phenotypes.

This study aims to investigate 338 CSF metabolites closely associated with the onset and progression of AD. By identifying metabolites causally linked to AD, we seek to understand their biological significance and their feasibility as biomarkers or therapeutic agents. Therefore, in this study, we employ two-sample MR to assess causal relationships between CSF metabolite levels and measures of general cognitive function (with three additional domains of the fluid intelligence score, prospective memory result, and reaction time) and measures of brain atrophy (cerebral cortical surface area and thickness and hippocampal volume).

## 2 Method

### 2.1 Study design

**Figure 1:**
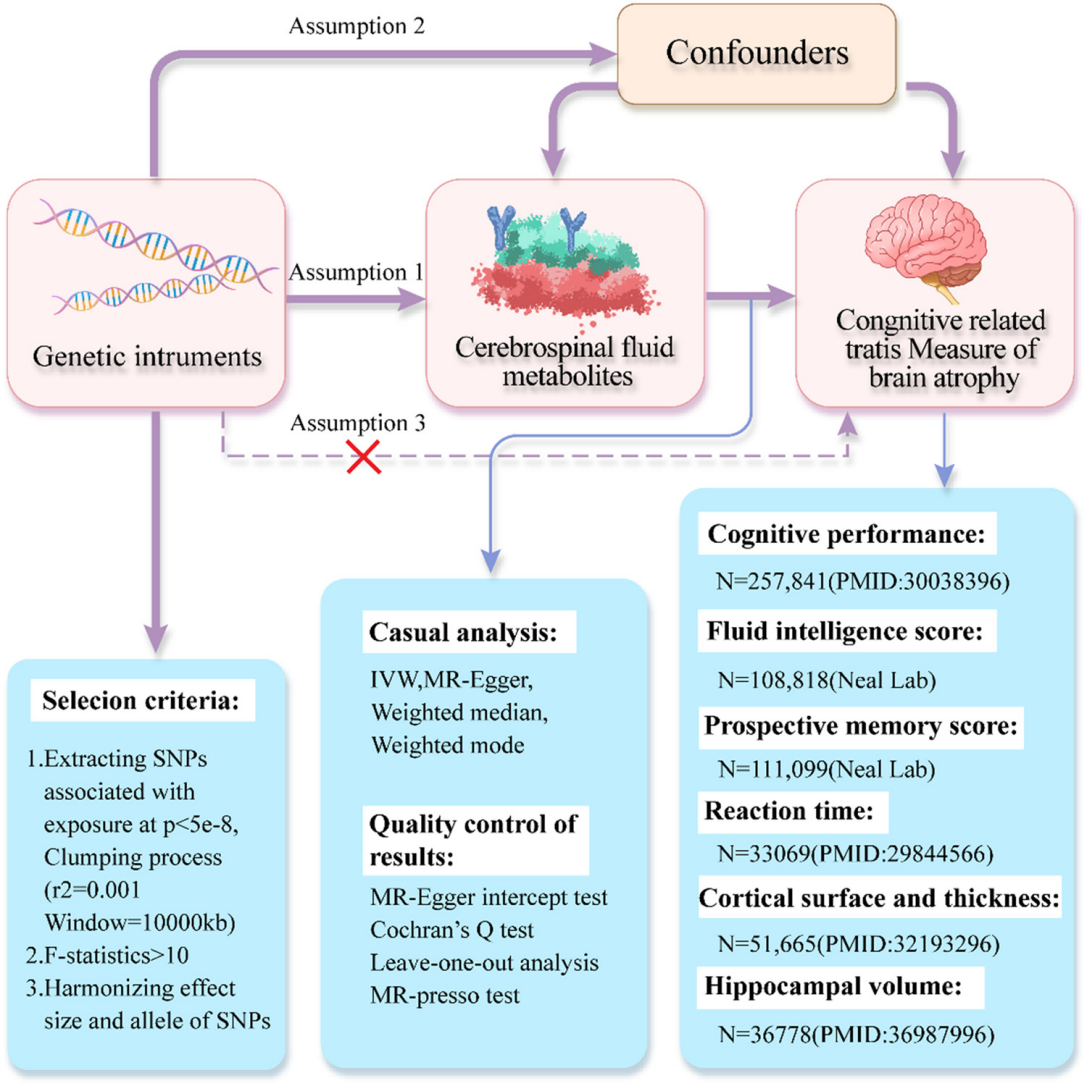
Overview of MR analysis. Assumption 1, genetic instruments are strongly associated with the exposures of interest; Assumption 2, genetic instruments are independent of confounding factors; Assumption 3, genetic instruments are not associated with outcome and affect outcome only via exposures. IVW, inverse variance weighted; MR-PRESSO, MR-Pleiotropy RESidual sum and outlier; SNPs, single-nucleotide polymorphisms.

We conducted a two-sample MR study, adhering to three hypotheses: (1) the IV is closely associated with the exposure of interest; (2) the IV is independent of confounding factors; and (3) the IV does not affect the outcome except through the exposure [15]. The data used in this study are summary statistics from genome-wide association studies (GWAS). The original studies obtained specific ethical approval and informed consent. The study design is illustrated in Figure 1.

### 2.2 Selection of IVs

Genetic instruments associated with 338 CSF metabolites were derived from recent GWAS studies. These studies performed genome-wide association analyses of 338 CSF metabolites in cognitively healthy participants with an average age of 60 years (data available for download from ftp://ftp.biostat.wisc.edu/pub/lu_group/Projects/MWAS/). Notably, this represents the most comprehensive report to date on genetic loci related to CSF metabolites, identifying nearly 1.8 million SNPs through genome-wide association scans and high-throughput metabolic profiling conducted by Panyard et al. [16]. The detailed names of the 338 metabolites are listed in Table S1, with metabolites named X having unknown chemical properties. Specifically, the study included 689 participants, of whom 532 were from the Wisconsin Alzheimer’s Disease Research Center (WADRC) and 168 were from the Wisconsin Registry for Alzheimer’s Prevention, each providing unique CSF samples for metabolite analysis. Selection criteria for WADRC participants included age ≥45 years, decision-making capacity, and ability to fast for 12 h, with exclusion criteria such as specific medical histories including renal dysfunction, congestive heart failure, and major neurological disorders (excluding dementia).

To meet hypothesis (1), we identified IVs related to blood metabolites through rigorous screening criteria from multiple perspectives. Given the moderate number of SNPs associated with metabolites, we selected a stringent significance threshold of *p* < 5 × 10^−8^ to choose SNPs associated with the metabolites. We used a *p*-value threshold of less than 5 × 10⁻⁸ for metabolite GWAS IVs, as suggested by Burgess and Thompson [26], to strengthen genetic effects, though this could exclude SNPs with minor associations. Subsequently, we aggregated SNPs by eliminating linkage disequilibrium (LD, *R* ^2^ > 0.001 and within 10,000 kb). To minimize weak instrument bias, we computed the *R* ^2^ and *F* statistics for each SNP. The *R* ^2^ and *F* statistics were calculated as follows:

\[{R}^{2}=\frac{2\times {\beta }^{2}\times {\mathrm{EAF}}\times (1-{\mathrm{EAF}})}{{[}2\times {\beta }^{2}\times (1-{\mathrm{EAF}})+2\times {({\mathrm{se}}(\beta ))}^{2}\times N\times {\mathrm{EAF}}\times (1-{\mathrm{EAF}})]},]\]

\[F=\frac{N-k-1}{k}\times \frac{{R}^{2}}{1-{R}^{2}}.]\]

Here, *β* represents the effect size of the target genetic variation, EAF denotes the allele frequency of the target genetic variation, se(β) indicates the standard error of the effect size of the target genetic variation, *R* ^2^ is the coefficient of determination for the IVs explaining the exposure level in the regression equation, *N* is the sample size of the exposure, and *k* is the number of SNPs (IVs). SNPs with *F* < 10 are defined as poor genetic variations and are excluded [17]. Next, we filtered SNPs associated with metabolites from the results (*p* < 5 × 10^−8^). We further matched SNPs for exposure and outcome, removing those with reverse effects or allele inconsistency (e.g., A/G versus A/C). Then, to satisfy hypothesis (3), we excluded SNPs from IVs associated with the outcome (*p* < 5 × 10^−5^). Finally, causal inference analysis was performed on metabolites with two or more SNPs [18].

### 2.3 Cognitive-related traits

Cognitive performance was evaluated using a meta-analysis weighted by sample size, utilizing data from the Cognitive Genomics Consortium (COGENT, *n* = 35,295) and the UK Biobank (*n* = 222,543), and extracting genetic association statistics pertaining to general cognitive performance from the most recent GWAS conducted on individuals of European ancestry (*N* = 257,841) [19]. Participants ranged in age from 16 to 102 years and had no history of stroke or dementia. In COGENT, cognitive performance was defined as the first unrotated principal component score derived from at least three different neuropsychological tests included in the study. In the UK Biobank, verbal-numerical reasoning (VNR) was assessed using 13 multiple-choice questions, with VNR scores reflecting the number of correctly answered questions within 2 min, designed to measure fluid intelligence. This assessment has been demonstrated to possess adequate reliability and validity [19,20].

GWAS data on fluid intelligence score (*N* = 149,051) and prospective memory score (*N* = 152,605) were obtained from the UK Biobank, evaluated using fluid intelligence questions and image tests, respectively [21]. Data for reaction time (*N* = 330,069) came from a study on genetic loci related to general cognitive function conducted by the Centre for Cognitive Ageing and Epidemiology at the University of Edinburgh [22]. This measure is based on the total time taken by the participants to correctly complete card-matching tasks.

### 2.4 Measurements of brain atrophy

Genetic associations with the cortical surface area (mm²) and thickness (mm) were identified using a genome-wide meta-analysis of brain *T*1-weighted magnetic resonance imaging (MRI) data from 51,665 healthy individuals of European ancestry aged 3.3–91.4 years across 60 cohorts, conducted by the Enhancing Neuro Imaging Genetics through Meta Analysis (ENIGMA) consortium [23]. Among these 60 cohorts, less than 1% of the participants had psychiatric disorders in the included case–control studies, with nearly all others being healthy. Cortical surface area was measured at the gray–white matter boundary, and thickness was defined as the average distance between the white matter and pial surface. The total surface area and mean thickness were calculated for each participant. Genetic associations were assessed using additive models within each cohort, adjusting for age, age squared, sex, interaction of sex with age and age squared, the first four multidimensional scaling components, diagnostic status (where applicable in case–control cohorts), and scanner-specific variables.

Genetic data for hippocampal volume were derived from a GWAS meta-analysis conducted by the ENIGMA and CHARGE consortia across 65 study centers, analyzing high-resolution brain MRI scans from 33,536 healthy individuals aged 11–98 years [24]. Bilateral average hippocampal volume (mm³) was defined as the mean volume of the left and right hippocampus. Genetic associations were evaluated within each study center, adjusting for age, age squared, sex, intracranial volume, four multidimensional scaling components, and diagnostic status. For studies involving data from multiple centers or scanners, adjustments were made for center effects, and mixed-effects models were used to account for familial relationships pertaining to family data.

### 2.5 Statistical analysis

Causal relationships between CSF metabolites and brain atrophy and cognitive decline were primarily assessed based on results from inverse variance weighting (IVW). The IVW estimates are derived from meta-analyses of the Wald ratio for all genetic variants [24]. IVW assumes no horizontal pleiotropy across all SNPs, providing the most accurate assessment of causal relationships [25]. Initially, we used IVW-based estimates to screen CSF metabolites causally linked to brain atrophy and cognitive decline. To ensure robust findings, we applied two additional methods to further evaluate metabolites with significant IVW estimates (*p* < 0.05). MR-Egger, weighted median (WM), and weighted mode methods were employed as complementary analyses. These methods offer more robust estimates under relaxed assumptions; WM allows for less than 50% of the SNPs to be invalid, while MR-Egger provides tests for horizontal pleiotropy and heterogeneity in the presence of all SNPs having horizontal pleiotropy [26]. Weighted mode considers each genetic variant’s contribution to causal inference more precisely, making it suitable for handling complex weight data [17]. The false discovery rate (FDR) correction was applied using a standard and well-established procedure to account for multiple comparisons and control the false discovery rate in statistical analyses.

Sensitivity analysis is crucial as it allows for examination of potential violations of horizontal pleiotropy and heterogeneity that could seriously bias MR estimates. Horizontal pleiotropy is observed when IV influences outcomes through pathways other than the exposure of interest. Therefore, several tests were conducted to ensure the credibility of estimates. In this study, we employed three methods to detect and correct for heterogeneity and pleiotropy, including Cochran’s *Q* test and MR-Egger intercept test. Heterogeneity was deemed present if Cochran’s *Q* test yielded a *p*-value <0.05 [27]. The MR-Egger intercept was calculated to assess directional pleiotropy and bias due to invalid IVs [26]. For robustness of results, we conducted leave-one-out (LOO) analysis, where each SNP was sequentially removed and MR analysis was rerun to evaluate the impact of individual SNPs [26].

To summarize, we systematically screened CSF metabolites potentially associated with brain atrophy and cognitive decline using several criteria: (1) significant *p*-values in the main analysis (*p* < 0.05 by IVW). (2) Consistent direction of beta values across four MR methods. (3) No heterogeneity or horizontal pleiotropy observed in MR results. (4) Minimal disruption of MR estimates by individual SNPs.

### 2.6 Reverse analysis

In order to investigate potential reverse causation, we also examined the relationships between genetically influenced cognitive performance and brain atrophy markers with 338 CSF metabolites. To achieve this, we extracted independent genetic variants significantly associated with cognitive performance, cortical surface area, and thickness, as well as hippocampal volume from the same GWAS dataset, utilizing these variants as IVs in our primary analyses. SNPs associated with the cortical surface area and thickness were excluded to mitigate potential pleiotropic effects. All analyses were performed using R (v3.6.3) statistical software (R Foundation for Statistical Computing, Vienna, Austria). MR analyses were conducted using the R package “TwoSample MR.” A *p*-value of 0.05 was considered statistically significant.


**Ethics and consent declarations:** This study utilized publicly available GWAS summary statistics and did not involve new human subject experimentation. Therefore, institutional ethics approval and individual participant consent were not required. The original studies obtained ethical approval in accordance with the Declaration of Helsinki.

## 3 Results

### 3.1 MR results

Following strict control of the IV P1 threshold (5e-8) and removal of linkage disequilibrium, we proceeded with further analysis using 228 out of the 338 metabolites, as outlined in Table S1. From the screened exposure-related SNP data, SNPs corresponding to the outcome were extracted to ensure analysis consistency and comparability based on the same SNP set. A threshold P2 (5e-5) was applied to exclude SNPs highly correlated with the outcome, aiming to mitigate confounding effects or spurious associations. SNP datasets demonstrating no reverse causation, identified through Steiger test results, were used to finalize the IVs for analysis (refer to Table S2). All F-statistics for SNPs associated with metabolites exceeded 10, confirming the strength of the IVs. Subsequent to IVW analysis, combined with complementary and sensitivity analyses, we pinpointed 30 metabolites potentially causally associated with brain atrophy and cognitive function.

**Figure 2:**
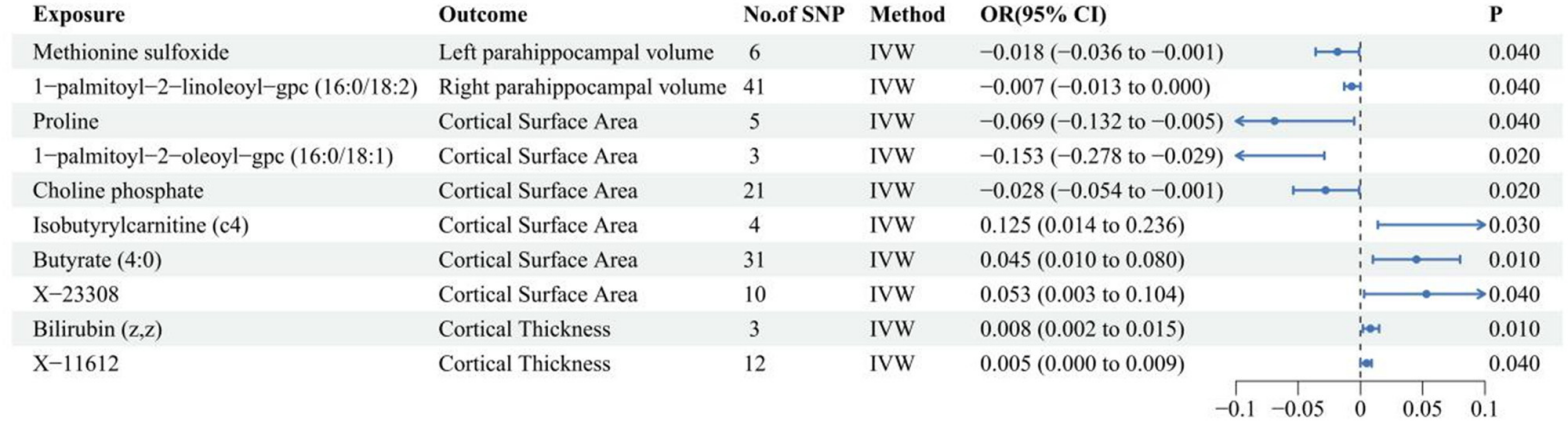
Association of CSF metabolites with measures of brain atrophy.

Ten metabolites meeting strict screening criteria are associated with brain atrophy, as shown in Figure 2. Methionine sulfoxide and 1-palmitoyl-2-linoleoyl-gpc (16:0/18:2) show increased levels correlated with smaller hippocampal volume. Proline, 1-palmitoyl-2-oleoyl-gpc (16:0/18:1), choline phosphate levels, isobutyrylcarnitine (c4), butyrate (4:0), and X-23308 are correlated with the cortical surface area. Increased bilirubin (*z*,*z*) and X-11612 levels are associated with thicker cortical thickness. Detailed results of all MR analyses for CSF metabolites and brain atrophy are presented in Table S3.

**Figure 3:**
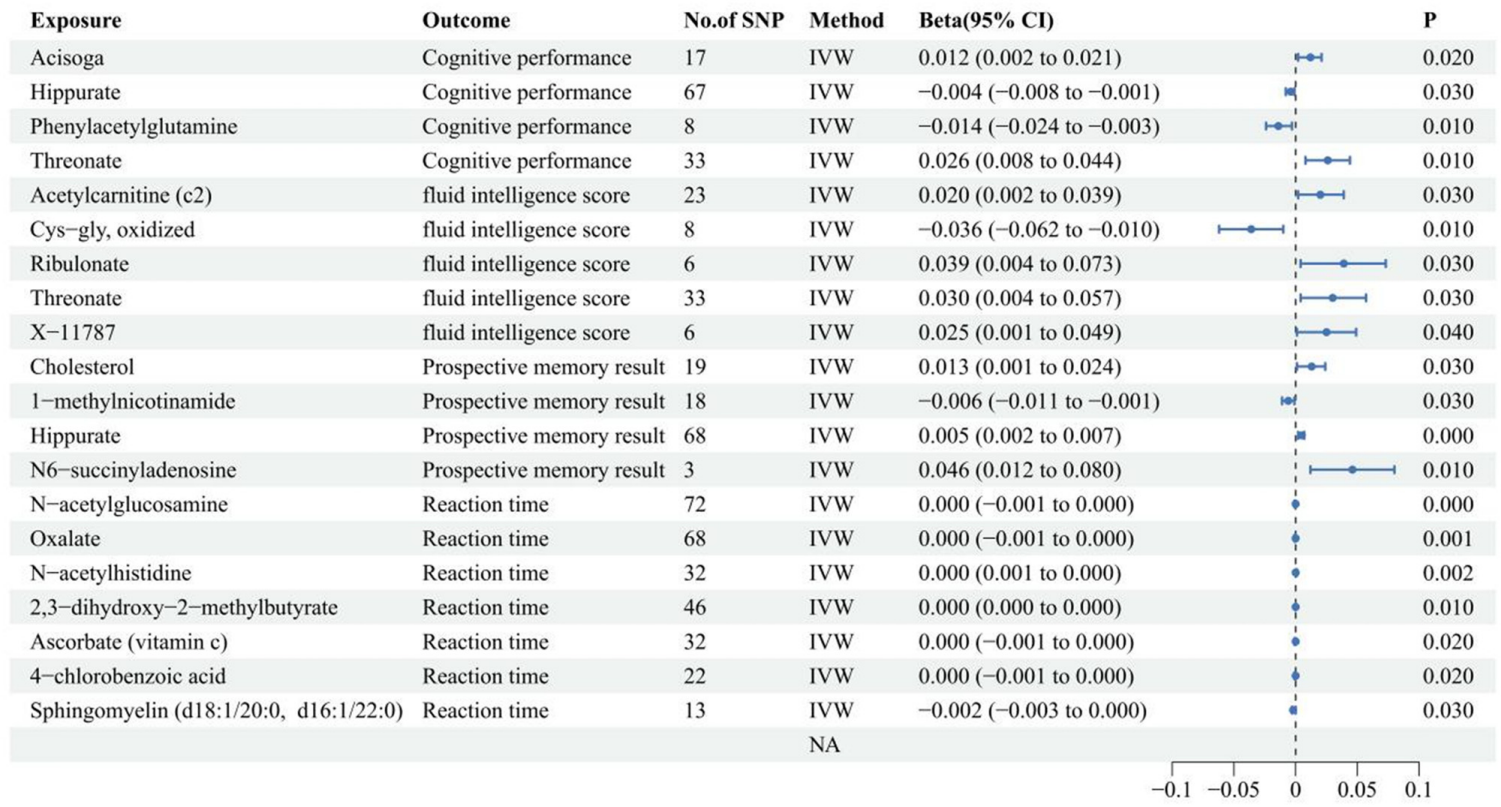
Association of CSF metabolites with measures of cognitive function.

Twenty metabolites meeting stringent screening criteria associated with cognitive function are detailed in Figure 3. Acisoga, hippurate, phenylacetylglutamine, and threonate are correlated with cognitive performance. Acetylcarnitine (c2), Cys-gly, oxidized, ribulonate, threonate, and X-11787 are associated with fluid intelligence scores. Cholesterol, 1-methylnicotinamide, hippurate, and *N*6-succinyladenosine are associated with prospective memory. *N*-Acetylglucosamine, oxalate, *N*-acetylhistidine, 2,3-dihydroxy-2-methylbutyrate, ascorbate, 4-chlorobenzoic acid, and sphingomyelin (d18:1/20:0, d16:1/22:0) are associated with the reaction time. Comprehensive results of all MR analyses on CSF metabolites and cognitive function are provided in Table S4. The substances causing these changes include *N*-acetylglucosamine and oxalate, both of which, after adjustment using FDR multiple comparisons, have adjusted *p*-values less than 0.05.

Thirty metabolites exhibited statistically significant estimated values from IVW (*p*  <  0.05), with consistent direction and magnitude of estimates across IVW, MR-Egger, and WM approaches (Tables S3 and S4). Cochran’s *Q* test (*p*  >  0.05) and MR-Egger intercept test (*p*  >  0.05) provided robust evidence against heterogeneity and pleiotropy (Tables 1 and 2). LOO analysis supported the absence of MR estimation bias due to individual SNPs (Figure S1).

**Table 1:** Heterogeneity and pleiotropy of CSF metabolites on brain atrophy

Exposure	Outcome	Heterogeneity test		Pleiotropy test	
		MR Egger Q	*P* value	MR-Egger intercept	*P* value
Methionine sulfoxide	Left parahippocampal volume	1.751	0.781	0.000	0.999
1-Palmitoyl-2-linoleoyl-gpc (16:0/18:2)	Right parahippocampal volume	50.357	0.105	−0.002	0.740
Proline	Cortical surface area	0.586	0.900	−0.001	0.940
1-Palmitoyl-2-oleoyl-gpc (16:0/18:1)	Cortical surface area	0.007	0.931	−0.006	0.892
Choline phosphate	Cortical surface area	14.510	0.753	0.016	0.369
Isobutyrylcarnitine (c4)	Cortical surface area	2.483	0.289	−0.002	0.929
Butyrate (4:0)	Cortical surface area	19.452	0.909	0.004	0.849
X-23308	Cortical surface area	7.579	0.476	−0.023	0.265
Bilirubin (z,z)	Cortical thickness	1.515	0.218	−0.001	0.811
X-11612	Cortical thickness	12.429	0.257	0.000	0.830

**Table 2:** Heterogeneity and pleiotropy of CSF metabolites on cognitive function

Exposure	Outcome	Heterogeneity test		Pleiotropy test	
		MR Egger *Q*	*P* value	MR-Egger intercept	*P* value
Acisoga	Cognitive performance	10.838	0.764	−0.014	0.146
Hippurate	Cognitive performance	76.364	0.158	−0.002	0.606
Phenylacetylglutamine	Cognitive performance	2.976	0.812	−0.007	0.110
Threonate	Cognitive performance	39.203	0.148	0.001	0.811
Acetylcarnitine (c2)	Fluid intelligence score	25.906	0.210	0.008	0.564
Cys-gly, oxidized	Fluid intelligence score	3.986	0.679	−0.001	0.938
Ribulonate	Fluid intelligence score	1.831	0.767	0.011	0.515
Threonate	Fluid intelligence score	40.427	0.120	−0.001	0.860
X-11787	Fluid intelligence score	3.518	0.475	0.003	0.814
Cholesterol	Prospective memory result	15.087	0.589	0.001	0.743
1-Methylnicotinamide	Prospective memory result	12.953	0.676	−0.002	0.756
Hippurate	Prospective memory result	58.792	0.723	0.000	0.942
*N*6-succinyladenosine	Prospective memory result	0.092	0.762	0.003	0.886
*N*-acetylglucosamine	Reaction time	11.947	1.000	0.000	0.964
Oxalate	Reaction time	18.912	1.000	0.000	0.352
*N*-acetylhistidine	Reaction time	3.346	1.000	−0.001	0.290
2,3-dihydroxy-2-methylbutyrate	Reaction time	13.532	1.000	0.000	0.552
Ascorbate (vitamin c)	Reaction time	8.217	1.000	0.000	0.958
4-Chlorobenzoic acid	Reaction time	6.364	0.998	0.000	0.525
Sphingomyelin (d18:1/20:0, d16:1/22:0)	Reaction time	0.725	1.000	0.000	0.729

### 3.2 Reverse analysis

In total, 147, 35, 42, 3, 12, 6, and 13 SNPs were, respectively, identified as associated with cognitive performance, reaction time, fluid intelligence score, prospective memory result, cortical surface area, cortical thickness, and hippocampal volume, which also appeared in CSF metabolite GWAS. Using the IVW method, changes in choline phosphate, hippurate, dimethylmalonic acid, homoarginine, citramalate, inosine, 3-hydroxy-3-methylglutarate, pyridoxate, myo-inositol, *N*-acetylglutamate, glycerol, argininate, isovalerate (i5:0), and X-12104 metabolites associated with cognitive performance were detected (Table S7). Comprehensive results of all MR analyses for cognitive performance and metabolites are presented in Table S8.

### 3.3 Metabolic pathway analysis

**Figure 4:**
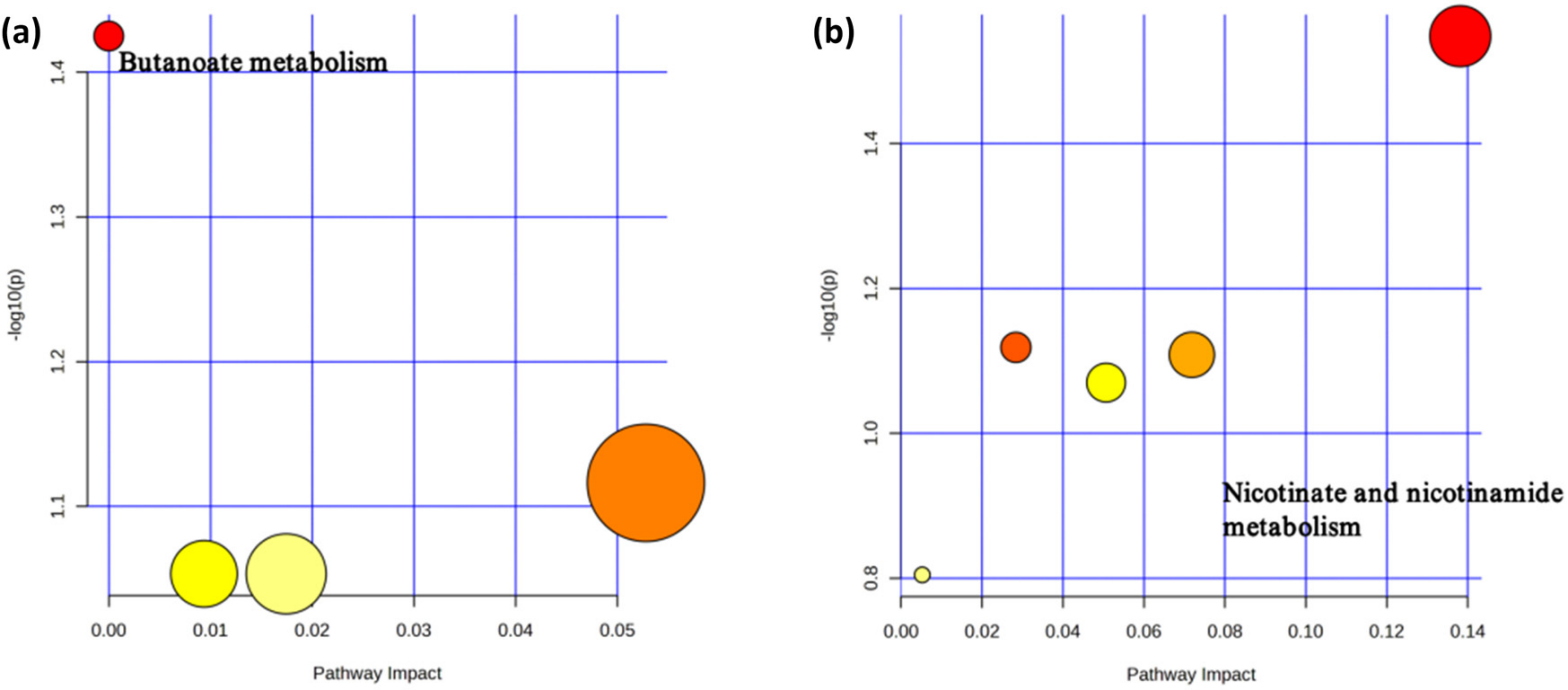
Potential metabolic pathways associated with brain atrophy and cognitive function. (a) Potential metabolic pathways involved in changes related to brain atrophy in MR positive analysis (based on metabolites). (b) Potential metabolic pathways involved in changes related to cognitive function in MR positive analysis (based on metabolites). Based on Kyoto Encyclopedia of Genes and Genomes (KEGG) pathway analysis. The color and size of each circle represent the *p*-value from topological analysis (yellow: higher *p*-value, red: lower *p*-value) and pathway impact score (larger circles indicate higher impact scores).

Metabolic pathway analysis based on metabolites identified two important metabolic pathways associated with brain atrophy and cognitive performance (Table S9). Our results indicate that “butanoate metabolism” (*p* = 0.0376) is associated with improvement in brain atrophy (Figure 4a), and “niacinamide and niacin ester metabolism” is associated with cognitive function (*p* = 0.0283) (Figure 4b).

## 4 Discussion

We identified 30 metabolites potentially and causally associated with brain atrophy and cognitive function, including 27 with known chemical compositions and 3 with unknown identities. The 27 known metabolites span categories such as amino acids, peptides, lipids, nucleic acids, vitamins, carbohydrates, and xenobiotics. Metabolic pathway analysis revealed two key pathways associated with brain atrophy and cognitive function: “butanoate metabolism” is linked to improvement in brain atrophy, and “niacinamide and niacin ester metabolism” is associated with cognitive performance. Additionally, there is a reverse causality between hippurate and cognitive performance.

The hippocampus is a crucial neural structure in the brain, particularly essential for learning and memory functions. Changes in its volume are typically associated with cognitive decline and neurodegenerative diseases such as AD. Generally, a reduction in the hippocampal volume is considered an early sign of cognitive decline. Our study found that increases in methionine sulfoxide and 1-palmitoyl-2-linoleoyl-gpc (16:0/18:2) are correlated with decreased hippocampal volume, consistent with the previous research findings. Methionine sulfoxide is an oxidized form of sulfur-containing amino acid, commonly associated with oxidative stress and antioxidant processes [28]. Methionine sulfoxide has also been reported to be closely related to the occurrence, development, and effective treatment of AD [29]. A specific phospholipid called 16:0/18:2, which is one of the main components of the cell membrane, may play an important role in cell signal transduction, lipid metabolism, and neuroprotection.

The cortical surface area of the brain refers to the total surface area of the outer gray matter, serving as a critical indicator of the brain structure and functional development. Studies indicate that the cortical surface area may be affected in AD patients, particularly in the early stages of the disease. Cortical atrophy and thinning are observed in AD patients, possibly linked to pathological processes such as neuronal loss, synaptic damage, and brain inflammation [30]. Proline primarily functions as an amino acid involved in protein structural stability and antioxidative effects in organisms, and is a neurotoxin associated with schizophrenia [31]. Our research has found a correlation between proline and the reduced cortical surface area, consistent with its detrimental role in neurodegenerative diseases. Isobutyrylcarnitine (C4), a short-chain acylcarnitine, is the most abundant carnitine in the human body. Studies suggest that levels of isobutyryl-l-carnitine in blood or plasma decrease in traumatic brain injury patients [32,33], consistent with our finding that lower levels of isobutyrylcarnitine (C4) correlate with smaller cortical surface area. Bilirubin’s antioxidant activity is particularly important for the brain, preventing excitotoxicity and neuronal death by clearing superoxide radicals during *N*-methyl-d-aspartate neurotransmission [34]. Similarly, our research correlates higher bilirubin levels with larger cortical surface area. Phospholipid 1-palmitoyl-2-oleoyl-gpc (16:0/18:1) also affects the cortical surface area by participating in the cell membrane structure and function. Choline phosphate is involved in phospholipid biosynthesis, impacting membrane stability and function. Butyric acid, a short-chain fatty acid, is metabolized in mitochondria through fatty acid metabolism as an energy source. Its role in the intestine and potential impact on overall health are supported by existing research, with lower butyric acid levels appearing correlated with multisystem atrophy [35]. Metabolomic pathway analysis reveals a significant association between butyric acid metabolism and brain atrophy, suggesting a potential beneficial role in neuroprotection and brain health via gut microbiota synthesis.

Cognitive performance, fluid intelligence scores, prospective memory outcomes, and reaction times are highly significant in studying cognitive functions, psychology, and neuroscience. Among these related CSF metabolites, acetylcarnitine (C2) participates in energy metabolism processes, potentially aiding in maintaining neuronal function and protecting the nervous system from damage [36]. Compounds such as Cys-gly, oxidized forms, and ascorbate (vitamin C) may benefit brain health through anti-oxidative and anti-inflammatory processes [37,38]. 1-Methylnicotinamide, the main metabolite of nicotinamide, has been reported to be associated with neuroprotection and can improve cognitive deficits [39]. Consistent with these findings, our results indicate that 1-methylnicotinamide leads to changes in prospective memory results. In studies of neuroprotection and cognitive function, threonate has shown significant advantages in improving cognitive abilities and memory [40]. Similarly, our research findings indicate that an increase in threonate enhances cognitive performance and fluid intelligence scores. Hippurate levels decrease in cognitive impairment disorders [41], which aligns with our finding that it alters cognitive performance and fluid intelligence scores. Phenylacetylglutamine and *N*-acetylhistidine are metabolites of amino acids. Ribulonate participates in the metabolic pathway of ribose. N6-Succinyladenosine is an adenosine analogue that may be involved in protein synthesis or energy metabolism pathways. Cholesterol and sphingomyelin (d18:1/20:0, d16:1/22:0) are lipids involved in the cell membrane structure and signal transduction. 4-Chlorobenzoic acid is a chlorinated benzoic acid used in chemical research or pharmaceutical synthesis. Oxalate participates in calcium metabolism and stone formation. These compounds have shown certain correlations with cognitive functions related to the brain in our research. Notably, two compounds remained significantly associated with the reaction time after FDR correction, highlighting their potential biological relevance. *N*-Acetylglucosamine, a biologically significant monosaccharide, plays a critical role in cellular processes, and its post-translational modifications may influence neuronal function and contribute to the pathogenesis of neurodegenerative diseases [42]. Oxalate, a naturally occurring organic compound primarily derived from dietary sources such as vegetables, legumes, and nuts, exhibits strong chelating properties and potential neurotoxicity. Consequently, tight regulation of its concentration in CSF may be essential to prevent neuronal damage [43]. In line with these findings, our results demonstrate that both *N*-acetylglucosamine and oxalate are negatively associated with reaction time, suggesting a potential link between these metabolites and cognitive processing speed.

In our reverse analysis, we found that only cognitive performance leads to changes in CSF metabolites, whereas other factors such as cortical area, thickness, hippocampal volume, fluid intelligence scores, brain cognition, prospective memory outcomes, and reaction times did not affect CSF metabolites. These two metabolites are worth noting. Elevated levels of hippurate are associated with the risk of age-related cognitive impairment, possibly due to an increase in hippurate following a high polyphenol diet, which enhances anti-inflammatory and antioxidative effects [41]. A high polyphenol diet appears to be the best dietary approach for preventing or delaying cognitive decline [44]. Additionally, from the perspective of gut microbiota, cognitive impairment significantly disrupts the composition and function of intestinal microbiota, where intestinal microbiota are involved in the synthesis and metabolism of hippurate [45]. Choline phosphate plays a crucial role in the synthesis of essential phospholipids for the human brain [46]. Higher levels of choline in brain metabolites are associated with the onset of psychiatric disorders [47]. Choline phosphate in the CSF is correlated with cognitive performance, though specific mechanisms require further research.

## 5 Advantages and limitations

To the best of our knowledge, this study represents the first comprehensive investigation of causal relationships between CSF metabolites and both cognitive function and structural brain atrophy. By employing a two-sample MR design (including bidirectional MR studies), we maximally avoided the pitfalls of reverse causation and residual confounding in observational studies. Second, this MR analysis extensively incorporates genetic variables, enabling us to make effective causal inferences. Finally, bidirectional MR design largely mitigates reverse causation and residual confounding factors [48]. However, several limitations need consideration in interpreting the results. First, the biological roles of certain identified metabolites and their associated pathways in cortical development and cognitive processes remain incompletely characterized, potentially constraining mechanistic interpretations of the MR findings. Second, as the analysis was restricted to European-ancestry populations, generalizability to other ethnic groups may be limited. Another limitation of the study is that the chemical properties of metabolites such as X-23308 and X-11612 remain unknown, which may affect the comprehensive interpretation of their roles and mechanisms.

## 6 Conclusion

In summary, this MR study establishes robust causal links between CSF metabolites and genetic determinants, while demonstrating their associations with cognitive function and structural brain metrics, including cortical and hippocampal volumes. These findings collectively underscore the potential role of CSF metabolites in modulating neurocognitive health. Future studies should prioritize multi-ethnic cohorts and longitudinal designs to strengthen causal inference, thereby advancing biomarker discovery for early diagnosis and disease monitoring in neurodegenerative disorders.

## Supplementary Material

Supplementary Figure

Supplementary Table
