# Supplementary material for: Exploring cerebrospinal fluid metabolites, cognitive function, and brain atrophy: Insights from Mendelian randomization
Source: Open Med (Wars). 2025 Aug 4;20(1):20251237. doi: 10.1515/med-2025-1237 (PMC12326306; doi:10.1515/med-2025-1237)
Supplement: Supplementary Figure [file med-2025-1237-sm.pdf]

Supplementary material

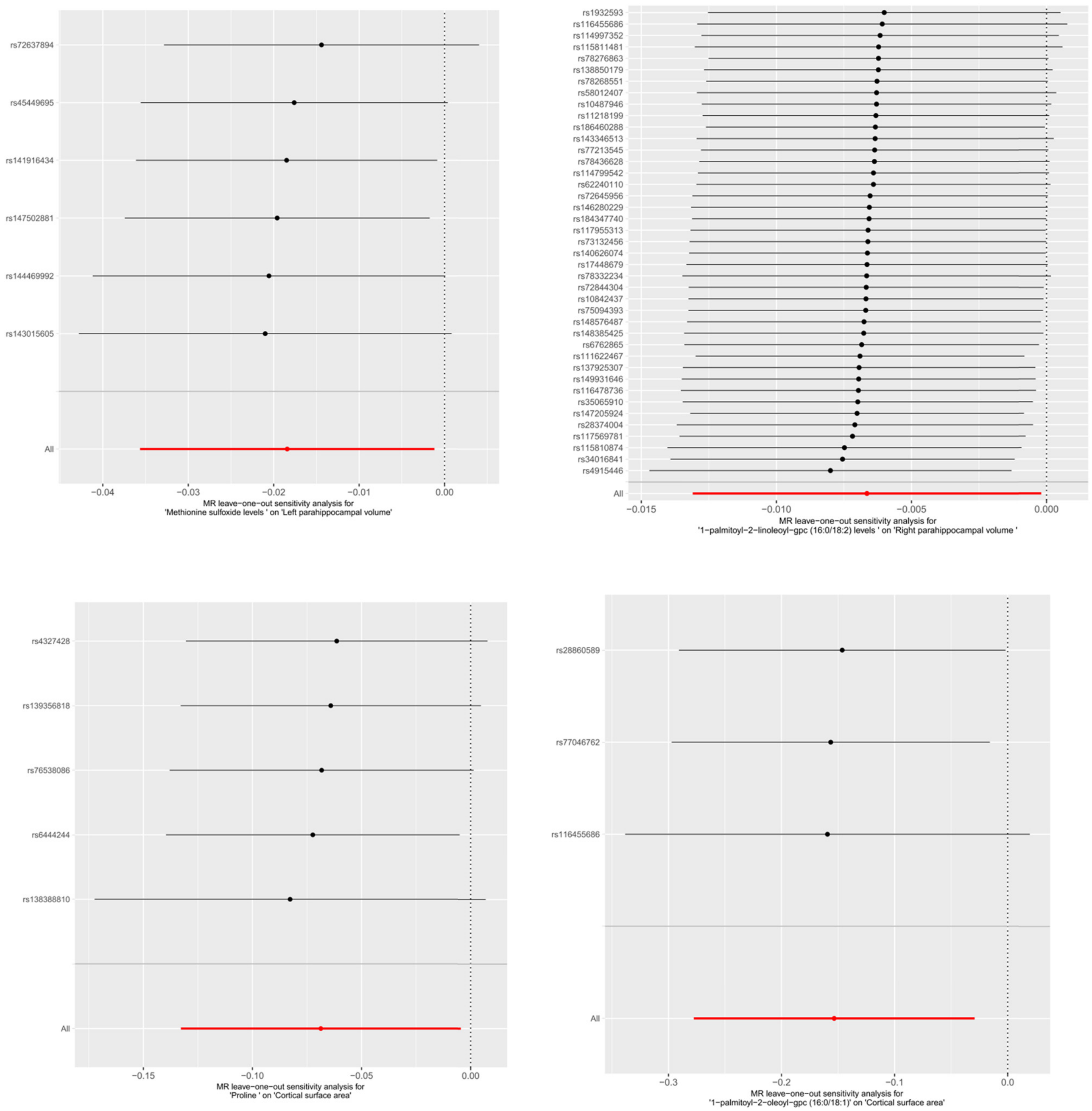

Figure S1: Forest plots for the Mendelian randomization leave-one-out analysis of the significant inverse variance weighted estimates.

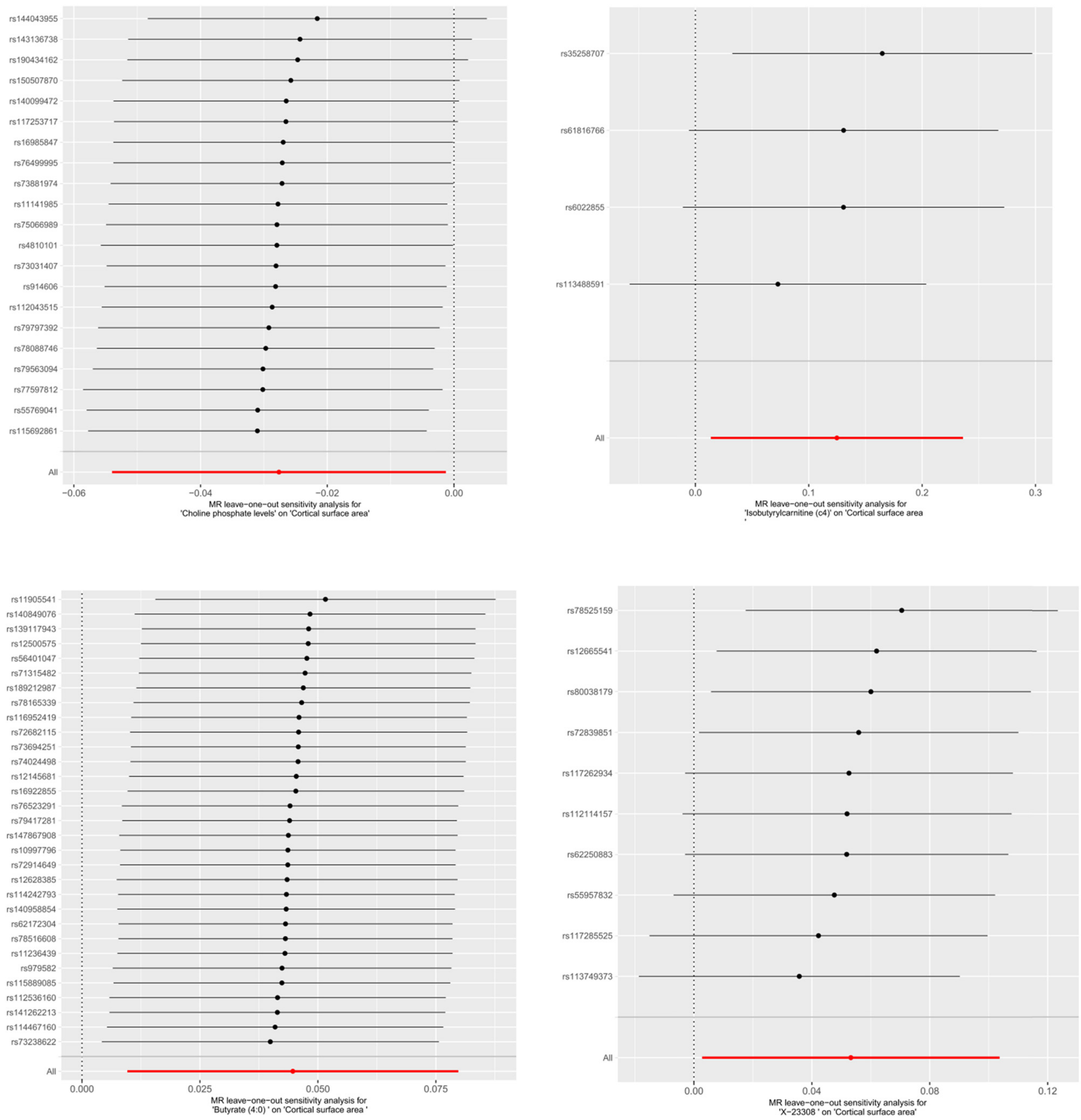

Figure S1: (*Continued*)

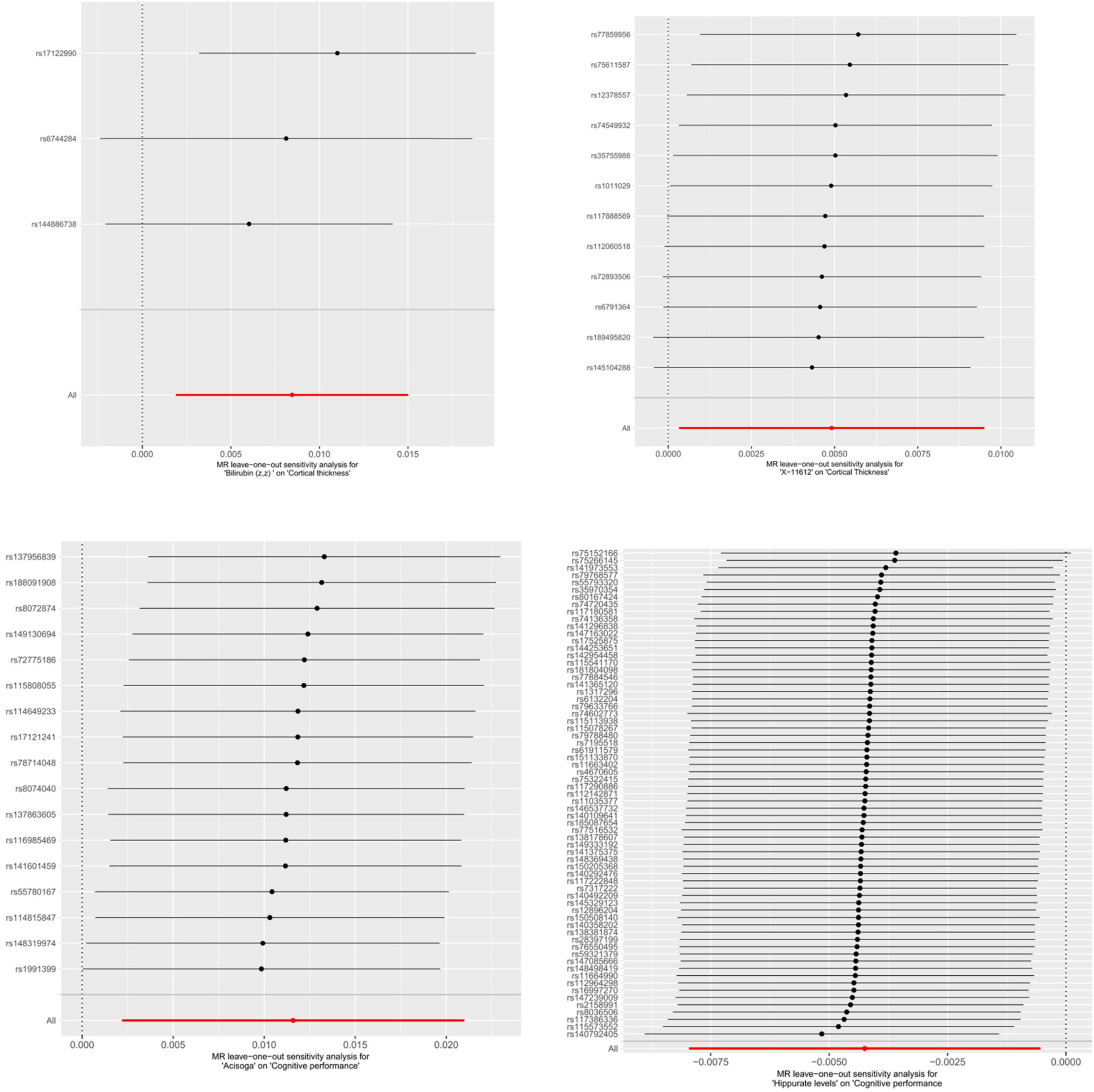

Figure S1: (*Continued*)

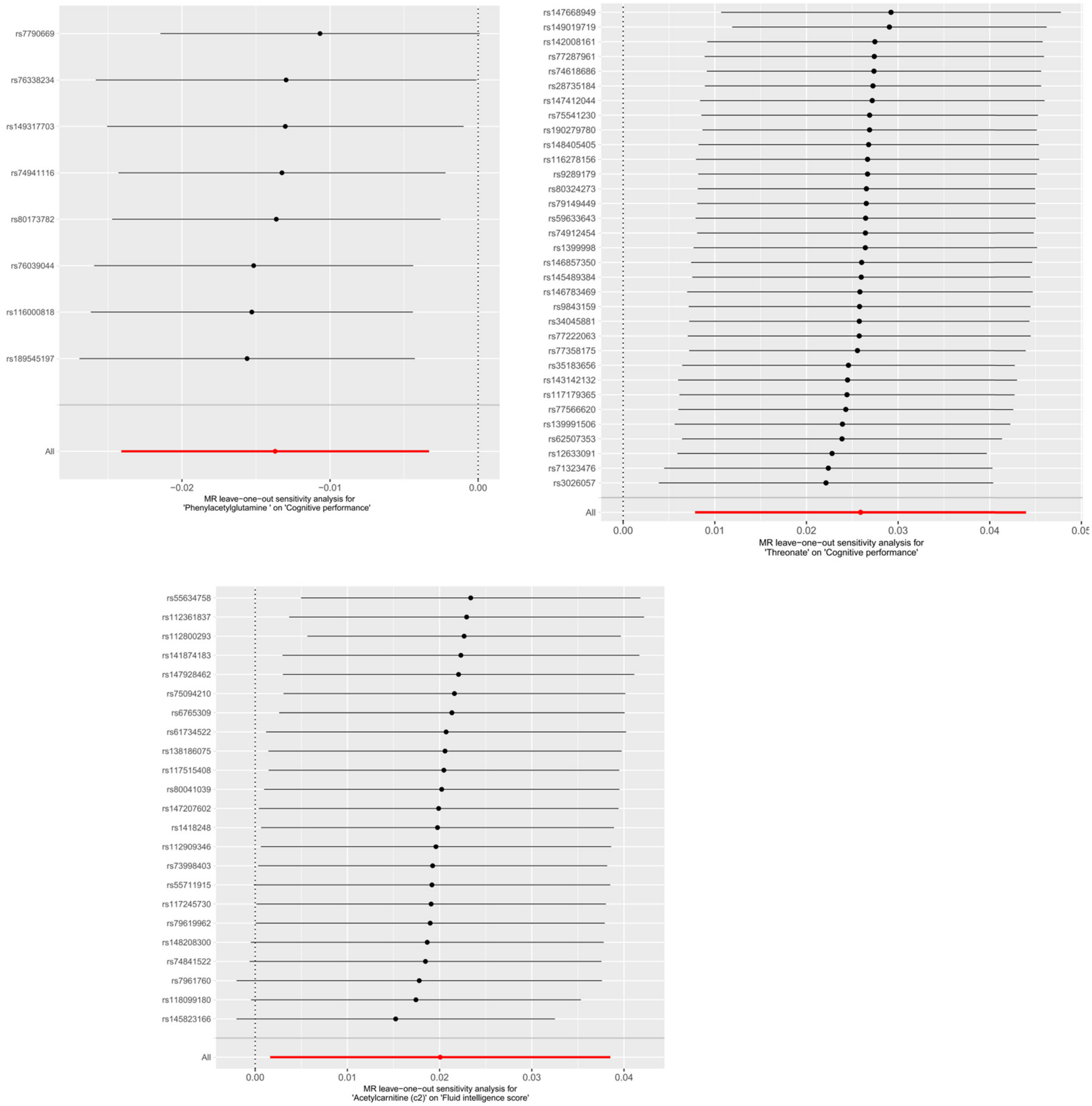

Figure S1: (*Continued*)

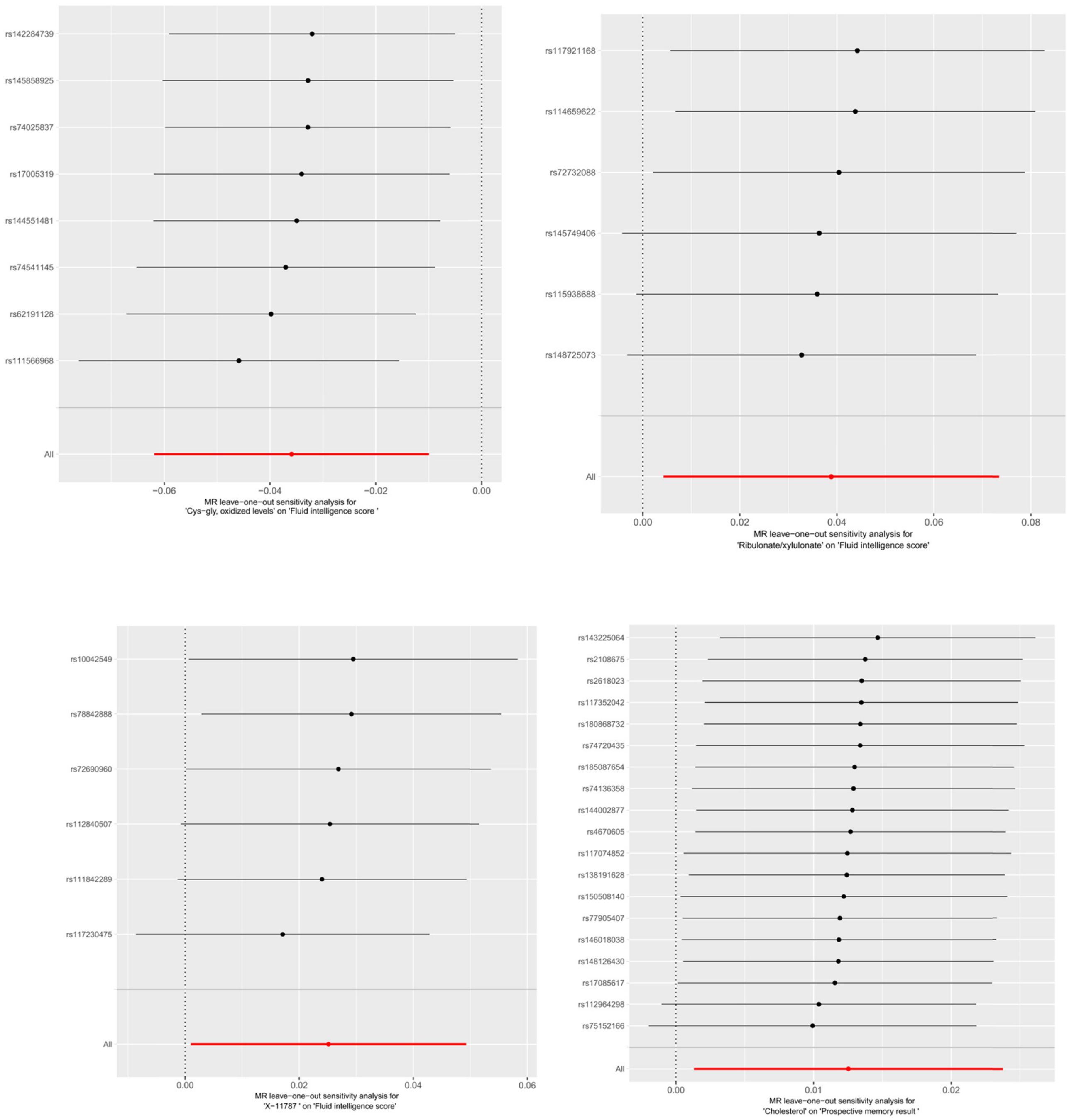

Figure S1: (*Continued*)

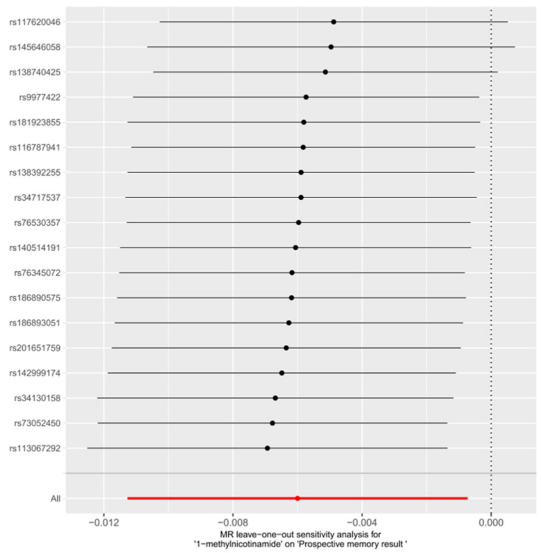

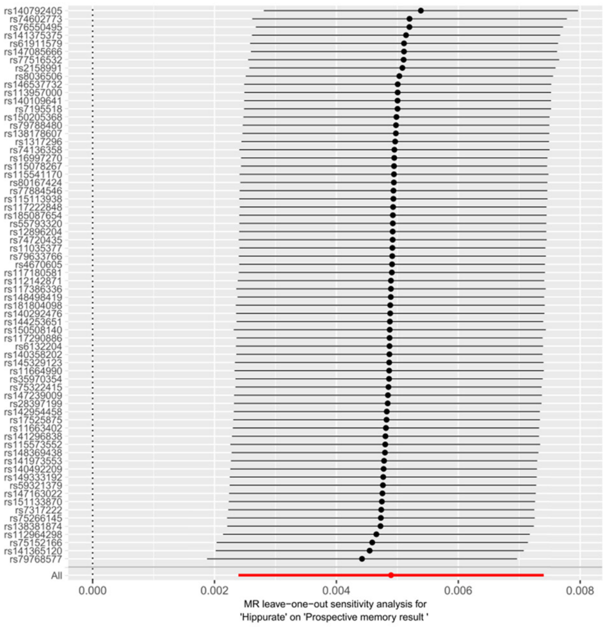

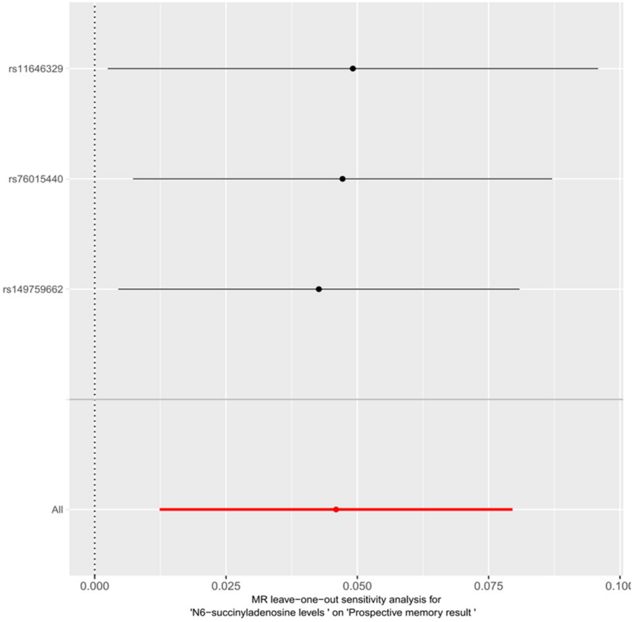

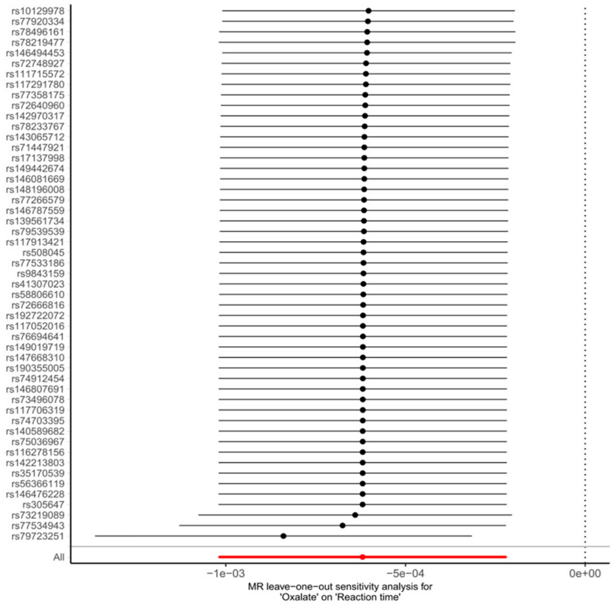

Figure S1: (*Continued*)

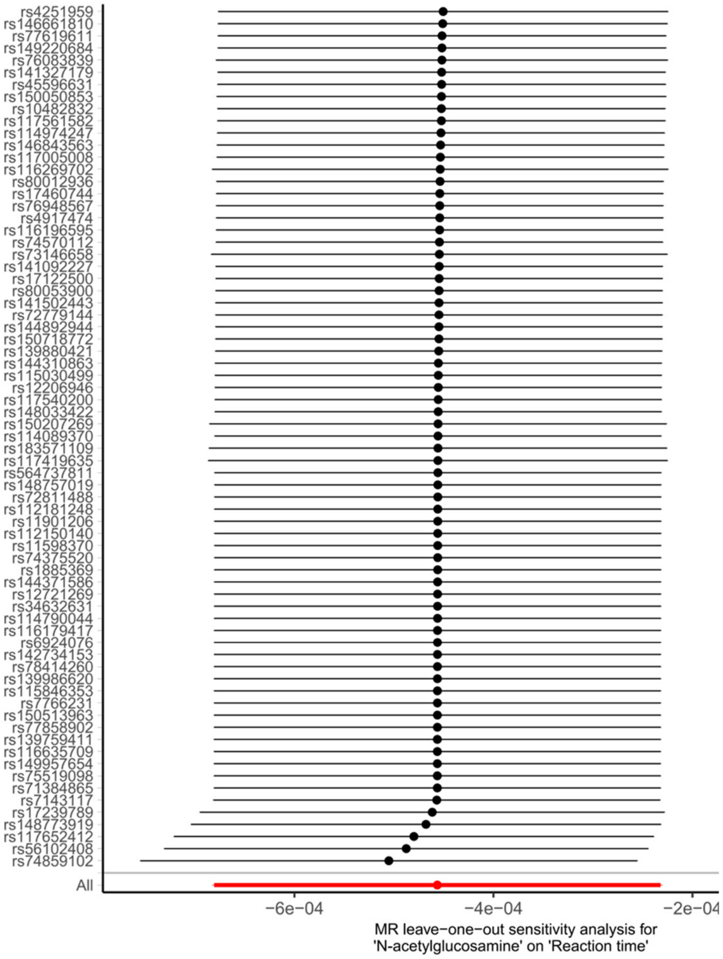

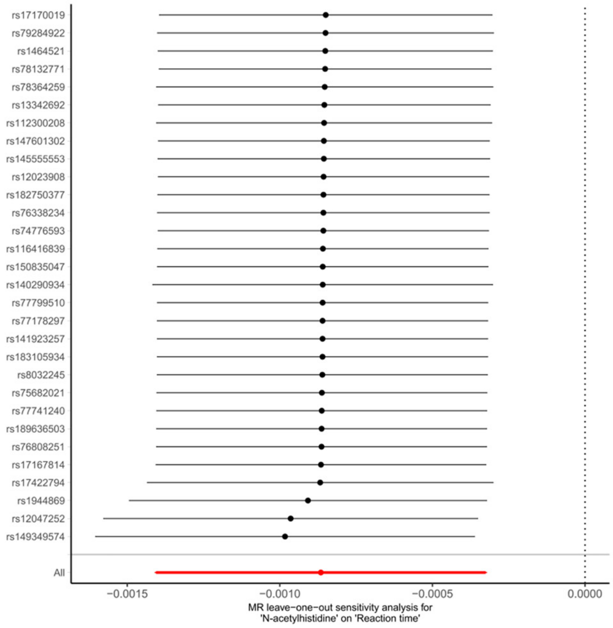

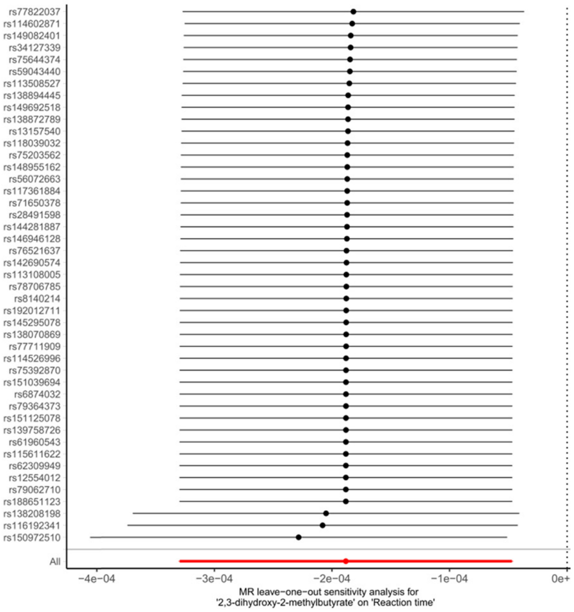

Figure S1: (*Continued*)

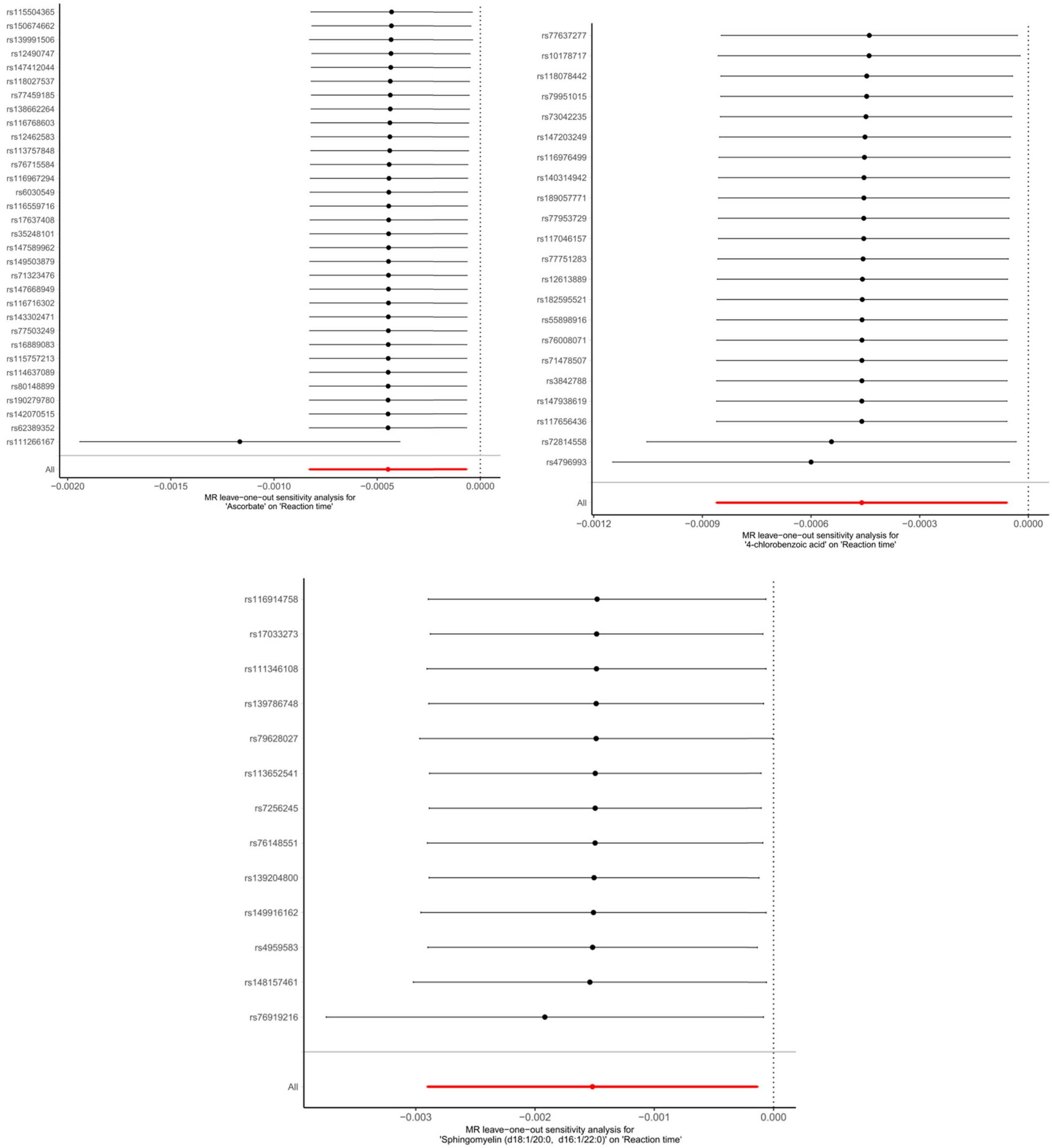

Figure S1: (*Continued*)
